# Invasive Aspergillosis in Pediatric Leukemia Patients: Prevention and Treatment

**DOI:** 10.3390/jof5010014

**Published:** 2019-02-11

**Authors:** Savvas Papachristou, Elias Iosifidis, Emmanuel Roilides

**Affiliations:** Infectious Diseases Unit, 3rd Department of Pediatrics, Faculty of Medicine, Aristotle University School of Health Sciences, Konstantinoupoleos 49, 54642 Thessaloniki, Greece; savvas.gpap@gmail.com (S.P.); iosifidish@gmail.com (E.I.)

**Keywords:** *Aspergillus*, anti-fungal agents, hematological malignancies

## Abstract

The purpose of this article is to review and update the strategies for prevention and treatment of invasive aspergillosis (IA) in pediatric patients with leukemia and in patients with hematopoietic stem cell transplantation. The major risk factors associated with IA will be described since their recognition constitutes the first step of prevention. The latter is further analyzed into chemoprophylaxis and non-pharmacologic approaches. Triazoles are the mainstay of anti-fungal prophylaxis while the other measures revolve around reducing exposure to mold spores. Three levels of treatment have been identified: (a) empiric, (b) pre-emptive, and (c) targeted treatment. Empiric is initiated in febrile neutropenic patients and uses mainly caspofungin and liposomal amphotericin B (LAMB). Pre-emptive is a diagnostic driven approach attempting to reduce unnecessary use of anti-fungals. Treatment targeted at proven or probable IA is age-dependent, with voriconazole and LAMB being the cornerstones in >2yrs and <2yrs age groups, respectively.

## 1. Introduction

Aspergillosis can be present in an acute or chronic form [1]. Syndromes of clinical significance include invasive aspergillosis (IA), chronic and saprophytic aspergillosis, and allergic aspergillosis [2]. We focus on IA because it occurs in immuno-compromised hosts. It is associated with notable morbidity and mortality in pediatric patients suffering from immuno-compromising conditions [3,4,5], with one multi-center retrospective study recording at a 52.5% mortality rate [6]. IA in children has also been related to increased financial costs [7]. While immuno-compromised pediatric patients also display susceptibility to invasive fungal disease (IFD), like IA, differences from adults have been highlighted to pertain to several aspects of these infections [8,9,10,11,12]. These differences are summarized in Table 1.

During recent years, clinicians have been extrapolating evidence from adult studies of IA, due to the lack of respective pediatric data [6]. According to the 2017 European Society for Clinical Microbiology and Infectious Diseases (ESCMID), the European Confederation of Medical Mycology (ECMM) and the European Respiratory Society (ERS) Joint Clinical Guidelines, according to recent guidelines by the Fourth European Conference on Infections in Leukemia (ECIL-4), pediatric recommendations about intervention are based on efficacy data from phase 2 and 3 trials in adults, on pediatric pharmacokinetic (PK), dosing, safety, supportive efficacy data, and on regulatory approvals [8,13].

In pediatric patients, risk factors for IA include primary immunodeficiencies and especially chronic granulomatous disease (CGD), secondary immunodeficiencies (associated with cancer chemotherapy and failure syndromes of the bone marrow), critical illness, chronic diseases of the airways, low birth-weight, and prematurity (the last two are related to neonatal patients) [3,4,5,14,15,16]. Additionally, immunosuppressive treatments—including corticosteroids in high doses and biologic agents interacting with immune pathways (like monoclonal antibodies targeting tumor necrosis factor alpha)—are also regarded as risk factors for IA [5,6,17]. Lastly, solid organ transplantation (SOT) is related to IA, which becomes more evident in the case of heart and/or lung recipients [1,18]. Nevertheless, the most significant risk factors are considered to be hematological malignancies and *hematopoietic stem cell transplantation* (HSCT) [1,6,19,20,21]. These two conditions constitute the two major risk factors that are commonly encountered in patients with IA [1,6,19,20,21]. Furthermore, the detection of IA in leukemia patients affects the decisions regarding the administration of chemotherapy [5,7]. More specifically, delayed delivery of chemotherapy decreases the risk for IA progression, on one hand, but, conversely, it renders the progression of the malignancy more likely [5]. This delicate balance makes it more urgent to address the management of this group of patients.

This article intends to review the current strategies for prevention and treatment of IA in pediatric leukemia patients. In the section of prevention, the following topics will be covered: (a) epidemiology and risk factors for IA in pediatric patients with leukemia, (b) anti-fungal prophylaxis, and (c) other preventive measures. Treatment will be subdivided into three main areas: (a) empiric treatment, (b) pre-emptive treatment, and (c) treatment for proven/probable IA. The latter will also include an analysis of the therapeutic approaches to invasive pulmonary aspergillosis (IPA) and the central nervous system (CNS) aspergillosis.

## 2. Prevention

### 2.1. Epidemiology and Risk Factors for Invasive Aspergillosis

The incidence of IA in pediatric patients with hematological malignancies has been estimated by several studies between 4.57% and 9.5% [7,20,22,23]. Identified routes of infection include the respiratory tract, the gastrointestinal tract, and the skin [24]. A retrospective multi-center study incorporating a diverse population [6] found lungs, skin, and paranasal sinuses as the most frequently affected foci of infection. Regarding microbiology, *Aspergillus fumigatus*, *Aspergillus flavus*, *Aspergillus terreus*, and *Aspergillus niger* were the predominant isolates (in order of frequency) in the previous study [6].

Recognizing pediatric patients with leukemia at risk for developing IA is the cornerstone of prevention. This will enable physicians to timely implement the appropriate strategies to reduce modifiable risk factors and initiate anti-fungal prophylaxis in pediatric leukemia and HSCT patients at high risk for invasive *Aspergillus* spp. [8]. Risk factors for IA in the previously mentioned pediatric patients are summarized in Table 2.

Generally, an IFD incidence >10% is considered high-risk [8]. Severe and persistent neutropenia, high-dose corticosteroid regimens, and damage to mucosal surfaces render these two groups of patients susceptible to IA [8,25,26]. A recent systematic review of publications since 1980, that addressed pediatric-specific factors for invasive fungal diseases (IFDs), indicated that increasing age is a risk factor in both groups [27]. In leukemia patients, the type of malignancy determines the risk, with acute myelogenous leukemia (AML) ranking first (3.7–28% risk), while relapse and de novo acute lymphoblastic leukemia (ALL) are associated with a 4–9% and a 0.6–2% risk for IA, respectively [1,20,21,28]. It should be noted, that according to other studies, the risk was nearly equal between AML and ALL patients [6], or even greater in ALL patients [7]. However, these observations could be attributed to the specific characteristics or limitations of the studies. Refractoriness among acute leukemia patients is also a significant risk factor for IA [2]. High-risk ALL is recognized as a risk factor, but the heterogeneity characterizing this group of patients was underlined by the International Pediatric Fever and Neutropenia Guideline Panel [27,29]. In HSCT recipients, an allogeneic transplant is associated with a greater risk for IA than an autologous one [2,30]. Specific risk factors in allogeneic HSCT include the development of graft-versus-host disease (GVHD), the extension of human leukocyte antigen (HLA) discordance, the presence of cytomegalovirus (CMV) or respiratory virus coinfection, and the colonization by *Aspergillus* spp. [1,28,31,32,33]. In addition, two strategies for reducing GVHD—T-cell depletion and CD34 selection—are also related to IA infection [2,32,34]. Despite the absence of a risk stratification model for IFDs in pediatrics, a differentiation between high-risk and low-risk patients has been attempted [27,29]. More specifically, AML, high-risk ALL, acute leukemia relapse, allogeneic HSCT, protracted granulocytopenia, and administration of corticosteroids in high doses are considered high-risk conditions [29]. All other conditions are low-risk [29]. Lastly, topics in the field of risk factors for further research include the role of lymphopenia in IFDs and IA and the development of a prediction model for IFDs [27].

Certain risk factors for IA in children with leukemia and HSCT are ward-associated. These include local epidemiology, environmental conditions, contamination of hospital water supply systems, and construction work [2,7,8,35,36,37,38,39].

### 2.2. Anti-Fungal Prophylaxis

Anti-fungal prophylaxis is divided into primary and secondary entities [8]. Primary is defined as the administration of antifungal agents to high-risk patients without infection, whereas secondary lacks a robust definition and occasionally coincides conceptually with treatment for proven/probable IFDs [8].

Initiation of primary prophylaxis for IA is justified due to the lack of efficacy of diagnostic tests and the dismal outcomes of this infection [5,13]. Anti-fungal agents used for primary prophylaxis include the triazoles itraconazole, voriconazole, and posaconazole, liposomal amphotericin B (LAMB) (in the systemic and aerosolized form) and the echinocandins micafungin and caspofungin. Fluconazole has no activity against molds and, thus, it is not used in prophylaxis against IA [8].

Itraconazole is active against both yeasts and molds and is administered at a per os (PO) dose of 2.5 mg/kg/12 h, provided that the patient’s age is ≥2 years [8], while therapeutic drug monitoring (TDM) is necessary to achieve the dosing target of ≥0.5 mg/L [40]. Itraconazole has been studied in pediatric cancer and HSCT patients and is considered a reliable option [41,42] even though prospective studies of larger scale are required to reach further conclusions [43]. The use of this azole is restricted by adverse reactions, according to a meta-analysis [44]. It is not approved in EU for patients <18 years of age [8].

Voriconazole has been found to be superior to itraconazole, in terms of tolerability, for allogeneic HSCT in patients ≥12 years. However, the two agents were equally effective in preventing IFD [45]. According to the ECIL-4, the recommended voriconazole dose in pediatrics for ages 2–<12 years, or 12–14 years with a body weight <50 kg, is 9 mg/kg/12 h for the PO forms and 9 mg/kg/12 h the first day, which is followed by 8 mg/kg/12 h on subsequent days for the intravenous (IV) forms [8]. For ages 12–14 years with a body weight ≥50 kg, or ages ≥15 years, the recommended dose for the PO form is 200 mg/12 h and, for the IV form, 6 mg/kg/12 h the first day, which is followed by 4 mg/kg/12 h on subsequent days [8]. In pediatric patients, voriconazole exposure displays substantial variability [46] and, consequently, TDM is required to maintain the plasma concentration of 1–5 mg/L [8,47,48]. In the “Voriproph” study—the largest cohort study of voriconazole chemoprophylaxis in children—the use of this agent has been well tolerated [49]. An age of <2 years is a contraindication to the use of voriconazole [8].

Posaconazole is approved for use as a chemoprophylactic agent with both anti-mold and anti-yeast activity in pediatric patients with AML, GVHD after HSCT and HSCT with a long neutropenic period [10,50,51,52]. Posaconazole is appropriate only for children ≥13 years, based on scant PK data from two adult clinical trials, which also recruited a few patients older than 13, but younger than 18 years [1,53,54]. This anti-fungal agent is available in two PO formulations including an oral suspension and gastro-resistant tablets [55]. For tablets, the established dose is 300 mg/24 h, whereas, for suspension, it is 200 mg/8 h [50,55]. A recent, non-randomized, single-center study in pediatric patients with HSCT found that the tablets were more reliable than the suspension in terms of plasma trough levels [55]. TDM is, however, still required, when the oral suspension is used, to maintain plasma levels ≥0.5 mg/L [56]. Use of posaconazole in patients <13 years of age is contraindicated due to the lack of PK data, unstable plasma concentrations, lack of an IV preparation, and undependable PO absorption [50].

Liposomal amphotericin B (LAMB), in various dosing regimens, has been assessed in several pediatric studies with positive results regarding its safety, efficacy, and feasibility [57,58,59]. The IV form is reserved for patients intolerant or with contraindications to the use of triazoles [13]. The recommended dosing scheme is either 1 mg/kg/48 h, or 2.5 mg/kg two times per week [8]. The aerosolized form of LAMB is described in ECIL-4 guidelines as prophylaxis against pulmonary infections. However, the route is not approved and doses in patients younger than 18 years have not been established [8].

Micafungin has been compared to fluconazole in a phase III randomized, double-blind clinical trial including both adults and children with HSCT-associated neutropenia [60]. The results were in favor of micafungin in terms of efficacy as a prophylactic agent [60]. However, the scarcity of pediatric data, the lack of PO forms, and the high cost restrict the use of micafungin in anti-fungal prophylaxis [50]. According to ECIL-4 guidelines, the recommended dose is 1 mg/kg/24 h or 50 mg if the patient’s weight ≥50 kg [8]. A dose of 2 mg/kg/24 h has been evaluated and found to be safe in children with allogeneic HSCT [61]. Micafungin is used in cases of intolerance or contra-indication to triazoles [13].

Caspofungin has also been evaluated in the setting of anti-fungal prophylaxis [50]. A randomized study including both adult and pediatric patients with AML and myelodysplastic syndrome (MDS) compared caspofungin to itraconazole and found similar efficiency and tolerability [62]. Studies including only pediatric patients are limited to two retrospective cohort studies [50,63,64]. The first compared caspofungin to LAMB in HSCT recipients and reported comparable efficiency [63]. The second study used micafungin as a comparator and recommended that caspofungin should not be preferred over micafungin [64]. Both studies recognized the need for the conduction of randomized clinical trials [63,64].

In summary, in high-risk, acute leukemia patients, the recommended agents for primary prophylaxis against IA include itraconazole, posaconazole, IV LAMB, aerosolized LAMB, micafungin, and voriconazole [8]. In allogeneic HSCT recipients, the options for IA prophylaxis are itraconazole or voriconazole, micafungin, LAMB, aerosolized LAMB, and posaconazole [8]. When GVHD develops, the recommended anti-fungals are posaconazole, voriconazole, itraconazole, IV LAMB, and micafungin [8]. All the above agents are ranked according to the strength of recommendation and quality of evidence and are summarized in Table 3. The grading system used is the one developed by the Infectious Diseases Society of America (IDSA) [8,65]. According to the previously mentioned system, each recommendation receives a letter (A, B, C, D, or E) reflecting its strength, followed by a Latin number (I, II or III) pertaining to the quality of evidence [65].

Secondary chemoprophylaxis, as mentioned above, is a vague term, which cannot be easily differentiated from continued treatment against previous IA [8]. It should be administered for the entire period during which the risk factors for IA persist (such as granulocytopenia and immunosuppression) and it should be targeted against the previous isolates of *Aspergillus* spp. [8,13]. The “VOSIFI Study” has evaluated voriconazole in the setting of allogeneic HSCT in adult patients and has found that this agent is an efficient option when considering secondary prophylaxis [66]. The regimen consisting of LAMB followed by voriconazole has been evaluated in pediatric patients with acute leukemia and IPA [67]. Other options include itraconazole and caspofungin. However, supporting evidence is derived from adult studies [8].

### 2.3. Other Preventive Measures

Apart from the administration of drug prophylaxis, several infection control strategies can be applied to prevent IA among pediatric leukemia and HSCT patients. These strategies aim to decrease the exposure to sources of mold spores, which would, otherwise, increase the risk of IA [1].

The cornerstone is the construction of a “protective environment” for inpatients, which regulates room ventilation and involves a specific number of air exchanges/hour, the application of high-efficiency particulate air (HEPA) filters (with or without laminar airflow), appropriate room sealing, automatic-closing doors, pressure monitoring (to maintain a positive pressure differential between the ward and the outside), and directed airflow [2,68]. Furthermore, plants and flowers are not permitted in these rooms, but the installation of shower filters is recommended [13]. In hospitals with a limited number of “protective environments,” strict criteria should be implemented regarding which patients will be accommodated in these wards [2,68]. Another option is the admission to a private ward with restricted connections [2].

In outpatients, the previously mentioned measures are not applicable and, thus, different recommendations have been developed for this patient group [69]. These include, among others, avoidance of construction areas, stagnant waters, and areas with increased moisture, avoidance of gardening and lawn mowing, appropriate checking of foods, and hand hygiene [69]. The efficacy against IA of surgical masks and N95 respirators has not been established [2].

In the case of construction or renovation works in the hospital, or in any adjacent sites, infection control strategies should be escalated and interdisciplinary committees should be established to ensure compliance with these strategies [68].

Environmental sampling for microbiological analysis is useful in cases of outbreaks even though its application as part of routine clinical practice has been questioned and there is a lack of data to support it [2,68]. Nevertheless, it is a useful tool to evaluate the function of the filters [13].

## 3. Treatment

### 3.1. Empiric Treatment

Empiric treatment should be started in individuals at high-risk for IA presenting with fever and neutropenia, which persist for a minimum of four days after the initiation of broad-spectrum anti-bacteria [1,8,29]. Another group for which empiric therapy is indicated includes neutropenic patients presenting with recurrent febrile episodes after defervescence following the administration of antibiotics [8]. This indication, however, has not been graded in the latest guidelines by ECIL-4. Moreover, ECIL-4 guidelines recommend initiation of empiric therapy in low-risk children with persistent fever accompanied by severe neutropenia and mucosal damage [8]. Nonetheless, according to the clinical practice guideline (CPG) developed by the International Pediatric Fever and Neutropenia Guideline Panel, empiric treatment should not be administered to low-risk pediatric patients with persistent fever and neutropenia [29]. This recommendation is supported by the results of a randomized, prospective study comparing either caspofungin or LAMB to no treatment in low-risk children [70]. In resource-poor settings with inadequate laboratory capabilities, empiric treatment has been associated with better results in individuals at high risk for IA [7].

The anti-fungal agents recommended for this approach are caspofungin and LAMB [8,29]. The first is administered at a loading dose of 70 mg/m^2^, which is followed by 50 mg/m^2^/24 h (maximum dose 70 mg/24 h) and the second at a dose of 1–3 mg/kg/24 h [8]. Use of these agents in pediatrics has been established by three randomized prospective trials [1]. The results of the first trial underlined the superiority of liposomal to deoxycholate amphotericin B (AMB) [1,71]. According to the second study, amphotericin B colloidal dispersion (ABCD) was superior to deoxycholate AMB in terms of adverse events, but similar in efficacy [1,72]. Lastly, the third study found that caspofungin performed similarly to LAMB in terms of efficiency and adverse effect rates [1,73]. Caspofungin at a dose of 50 mg/m^2^/24 h in pediatric patients results in similar exposure levels with adult patients [74]. The use of voriconazole in empiric treatment has also been studied in a randomized, multi-center, open-label trial that compared this second-generation azole with LAMB [2,75]. The trial included both adult and pediatric patients and the results were indicative of a comparable response rate between voriconazole and LAMB groups in high-risk patients [2,75]. In another study, though, oral voriconazole was not preferred over deoxycholate AMB in patients presenting with gastrointestinal symptoms or in those receiving vincristine [7]. Identification of new anti-fungals to be used in empiric treatment remains a “research gap” [29].

Lastly, the duration of empiric anti-fungal schemes is a field that requires further investigation [29]. The 2017 ESCMID-ECMM-ERS Joint Clinical Guidelines recommend that the administration of caspofungin or LAMB should be carried on until defervescence and recovery of the neutrophil count [13,71,73,76].

### 3.2. Pre-Emptive Treatment

Empiric treatment has the disadvantage of exposing most patients to unnecessary use of anti-fungal drugs, due to the low specificity of fever as a symptom of IA [2]. Furthermore, the low sensitivity of fever as a criterion in the diagnosis of IA might delay the initiation of treatment. On the other hand, establishing a definite diagnosis in these patients is challenging and the risk of a dismal outcome is high [5,77]. Thus, the need for the adoption of a pre-emptive approach is highlighted [5,77].

Pre-emptive treatment is an alternative to empiric treatment that uses clinical and noninvasive, non-culture diagnostic methods to further assess the risk of IFDs and IA in febrile patients with neutropenia or in asymptomatic patients [2,8]. The diagnostic methods utilized in this approach include imaging techniques—mainly computed tomography—and microbiological markers, such as galactomannan (GM) antigen, (1,3)-β-d glucan (BDG), and *Aspergillus* polymerase chain reaction (PCR) [2,8].

Data regarding the application of pre-emptive therapy and the use of the associated biomarkers in children are limited [2,8]. Furthermore, these biomarkers do not perform optimally and consistently in pediatrics [78]. More specifically, the GM antigen displays variable sensitivity and specificity, low positive prognostic value (PPV), and a high false-positive rate [78,79]. The high negative prognostic value (NPV) of this biomarker applies only for *Aspergillus* spp. and not for other pathogens [78,79]. The sensitivity and specificity of BDG ranged from 50% to 82% and from 46% to 82%, respectively [78]. According to the International Pediatric Fever and Neutropenia Guideline Panel, its use is not recommended in the context of empiric anti-fungal treatment [29]. Lastly, the lack of standardization and the false-positive rates limit the use of PCR as well [78]. According to ECIL-4 guidelines, however, there is consensus in the favor of the use of the pre-emptive approach in pediatric patients, which has not received any grading [8]. A recent randomized, multi-center study compared pre-emptive with empiric treatment in pediatric cancer patients with fever and neutropenia with the exception of HSCT recipients [80]. The two methods were found to be comparable in terms of efficiency [80].

### 3.3. Targeted Treatment for Proven/Probable Invasive Aspergillosis

The European Organization for Research and Treatment of Cancer/Invasive Fungal Infections Cooperative Group (EORTC) and the National Institute of Allergy and Infectious Diseases Mycoses Study Group (MSG) provided the definitions for proven and probable IA. Proven IA requires evidence of *Aspergillus* to be identified in tissue by microscopic examination or culture, whereas probable entails a combination of patient risk factors, clinical manifestations, and mycological criteria [77]. Nevertheless, establishing a diagnosis is a challenging and time-consuming task, which may postpone treatment initiation and, thus, the above definitions should be used only for studies and not for routine clinical practice [13,77]. In the current review, the definitions by EORTC/MSG are used.

Targeted treatment for IA includes anti-fungal medications and adjunctive measures [2]. Similarities between pediatric and adult patients do exist, but there are several critical differences, such as the dosing scheme [2]. Targeted anti-fungal drugs (summarized in Table 4) can be further divided into the first and second-line, with the latter being reserved for unresponsive patients or for cases of intolerance to the adverse events [1,8]. Strength of recommendation and quality of evidence are in line with the IDSA grading system [8,65].

#### 3.3.1. First-Line Anti-Fungal Drugs

First-line agents are voriconazole, LAMB, and amphotericin B lipid complex (ABLC) [1,8].

Adequate data and experience have rendered voriconazole the cornerstone of IA treatment in children of all ages, apart from neonates and children <2 years [2,13]. A randomized clinical trial underlying its superiority against deoxycholate AMB in patients ≥12 years of age has played a significant role in establishing the use of this azole [83,84,85]. The PK of voriconazole is linear in pediatric patients, in contrast to the nonlinear pattern observed in adults and further population analyses of this parameter have facilitated the development of the dosing scheme [2,86,87]. For ages 2–<12 years, or 12–14 years with a body weight <50 kg, the dose is 9 mg/kg/12 h for the PO forms and 9 mg/kg/12 h the first day, followed by 8 mg/kg/12 h on subsequent days for the intravenous (IV) forms [8]. For ages 12–14 years with a body weight ≥50 kg, or ages ≥15 years, it is 200 mg/12 h for the PO form and for the IV form 6 mg/kg/12 h the first day, followed by 4 mg/kg/12 h on subsequent days [8]. According to a meta-analysis that highlighted the value of voriconazole TDM, therapeutic plasma levels increased the probability of a successful result, whereas greater concentrations were likely to cause toxicity [85,88]. TDM is, therefore, recommended, with an optimal range of 1–5 mg/L [8,47,48].

Liposomal amphotericin B (LAMB) is recommended at a dose of 3 mg/kg/24 h, IV [8]. The latter dose has been compared to a regimen of 10 mg/kg/24 h in the “AmBiLoad trial.” However, the higher dose has not been associated with greater efficacy [89]. Moreover, in the previous randomized trial, no direct comparison to voriconazole has been attempted [13,89]. LAMB is indicated predominantly for children <2 years and neonates, for whom voriconazole has not been approved, and for settings with increased prevalence of azole-resistance [1,13]. When compared to deoxycholate AMB, LAMB is less nephrotoxic and has fewer infusion-related toxic reactions, but its use is limited due to its high cost [90,91,92].

Lastly, ABLC is another option for the first-line targeted treatment of IA, which is administered at 5 mg/kg/24 h in one IV dose [8]. However, recommendations for the use of ABLC stem from experience in naïve patients who received the agent in terms of second-line therapy [8].

#### 3.3.2. Second-Line Anti-Fungal Drugs

Second-line agents include caspofungin, micafungin, itraconazole, and posaconazole [8]. The anti-fungal drugs described in the section of first-line treatment may also be used [8]. Voriconazole is reserved for patients naive to this agent and LAMB is used in cases of unresponsiveness or intolerance to the former, as well as in patients naive to AMB [1,8].

Caspofungin is the most preferred echinocandin for pediatric IFDs, based on results from the Antibiotic Resistance and Prescribing in European Children (“ARPEC”) study [50,93]. The recommended IV dose is calculated, according to the body surface area (BSA), and is defined as a 70 mg/m^2^ loading dose during the first day, which is followed by 50 mg/m^2^/24 h on subsequent days (maximum 70 mg) [2,8,74]. Caspofungin is approved for pediatric use both in the United States of America (USA), by the Food and Drug Administration (FDA), and in Europe [2,8]. Two prospective studies verified the efficacy and safety of caspofungin [50]. The first evaluated the drug in the setting of salvage therapy for IA with encouraging results that showed 45% of patients demonstrated complete or partial clinical response [94]. The second study showed that the use of caspofungin in children from 6 months to 17 years of age was efficient against IA in a consistent manner with adult studies [95]. Lastly, caspofungin has been confirmed to be a feasible alternative choice for the treatment of children with IFDs, based on results from a systematic review and meta-analysis [96]. The authors, though, acknowledge the need for further research on this topic [96].

Micafungin—for therapeutic purposes—is administered at a dose of 2 to 4 mg/kg/24 h, IV, which reached a maximum of 100 to 200 mg/24 h if the patient weights ≥50 kg [1,8]. ECIL-4 stated that its use as a second-line agent for IA is a non-approved indication, due to a lack of robust evidence [8]. The FDA has approved the use of micafungin in pediatric patients ≥4 months of age [2]. Dose adjustments need to be considered in children ≤8 years, due to the fact that micafungin clearance increases with decreasing age, which necessitates higher doses in young age groups [2,97]. When compared to triazoles in a meta-analysis evaluating the treatment of IFDs in hematologic patients with neutropenia, micafungin has been associated with higher efficacy, fewer severe adverse events, but displayed similar all-cause mortality [50,98].

Itraconazole, despite being the first in its class exhibiting anti-Aspergillus activity, has fallen into disuse, due to several limitations including unpredictable bioavailability, interactions with chemotherapeutic drugs such as cyclophosphamide, and interactions with drugs that cause QTc prolongation [2,82,85,99]. Using this azole as second-line therapy is not approved in individuals <18 years of age [8] and it is reserved for less critical cases of IA [2]. When used in patients ≥2 years old, the dose of the PO form of itraconazole is 2.5 mg/kg/12 h with subsequent TDM, to aim for plasma concentrations ≥0.5mg/L [1,8,40].

Posaconazole exists in three different formulations: oral suspension, gastro-resistant tablet, and IV solution [85]. It is not approved in the European Union (EU) for children <18 years of age [8], but has received approval by the FDA for patients ≥13 years for both PO formulations and for patients ≥18 years for the IV form [2]. Based on limited PK data in pediatric patients ≥13 years of age, the dose of oral suspension of posaconazole is 800 mg/24 h divided in 2 to 4 doses [1,8]. TDM is required to maintain plasma concentrations ≥0.7–1.5 mg/L [8,56,100]. No need for TDM exists for the PO gastro-resistant tablets [50]. Posaconazole may be used for salvage therapy in cases of refractoriness or intolerance to previous agents among pediatric patients aged 13 years or older [8,84,85,100].

It should be noted that treatment for proven/probable IA in pediatrics is age-dependent. More specifically, the options for children ≥2 years of age are voriconazole, LAMB, ABLC, ABCD, caspofungin, itraconazole, and posaconazole (for ages ≥13 years), whereas, for children <2 years old, LAMB, ABLC, and caspofungin may be used [5,13]. For neonates, the only option is LAMB [13].

#### 3.3.3. Novel Anti-Fungal Drugs

A brief review has to be made regarding the role that the newer agents of echinocandins and triazoles play in treating IA. These drugs are anidulafungin and isavuconazole, respectively.

Anidulafungin has not received FDA approval yet for use in patients aged <18 years [2,50]. The safety and PK parameters of anidulafungin in neutropenic pediatric patients at risk for IA have been assessed in a multi-center, dose-escalation study [101]. Anidulafungin displayed good tolerability and the regimen of 3 mg/kg loading dose, which was followed by 1.5 mg/kg/24 h that resulted in concentration profiles consistent with the adult dose of 100 mg/24 hours. This is the preferred one for IA [101]. The same regimen has been recently evaluated in a single-center study from Argentina, which underlined the safety and efficiency of this agent [102]. Lastly, the results of an open-label, non-comparative, pediatric study for the use of anidulafungin in invasive candidiasis (IC) have also been in favor of the safety of this drug in children, when administered in the previously mentioned doses [50,103].

Isavuconazole is a novel triazole with an extended spectrum of activity [104]. Its use in adult patients has been established by the “SECURE” trial—a phase 3, multi-center, randomized, double-blind, trial—which highlighted the safety and the non-inferiority of isavuconazole compared to voriconazole for the treatment of IA [85,105]. In pediatric patients, the PK and safety of both oral and IV forms of isavuconazole are currently being evaluated by a phase 1, open-label, multi-center study against no comparators (ClinicalTrials.gov NCT03241550) [50]. Additionally, limited experience has also been reported regarding the successful use of isavuconazole in pediatric hematology-oncology patients with invasive mucormycosis [50,106].

#### 3.3.4. Combination Therapies and Duration of Treatment

The role of combination therapies in pediatric cancer and HSCT patients with IA has not been completely elucidated and requires further assessment [5,85]. However, use of a combination of anti-fungals in such patients has been reported by two multi-center cohort studies [85] including one retrospective [6] and one prospective [107]. Moreover, ECIL-4 provides a recommendation for the use of such therapy—both in the setting of first and second-line treatment for IA—but this is based on poor evidence [8]. Safety and efficacy data have arisen from a study evaluating combination therapy with caspofungin in 40 hematologic pediatric patients with IA [8,108]. In both ECIL-4 and IDSA 2016 Guidelines, the recommended combination is that of a polyene or triazole with an echinocandin [2,8].

There is no consensus regarding the optimal duration for which patients with proven/probable IA need to be treated and, thus, this parameter should be individualized [5,8]. Duration of treatment has been documented to range from 3 to more than 50 weeks [13,83,89,105,109]. Treatment may be discontinued after clinical improvement, microbiological response, and recovery from GVHD [5,8,13].

#### 3.3.5. Breakthrough Infection and *Aspergillus* Resistance

Treatment of breakthrough infection occurring in patients who have received anti-mold prophylaxis includes salvage therapy, changing the antifungal drug class, awareness regarding local epidemiology patterns, and verification of serum triazole levels [2]. Salvage therapy is also used for the refractory or progressive aspergillosis and involves a switch in the class of antifungals, the addition of a second agent, the correction of underlying immunosuppression, and surgery [2,5].

Another essential topic that needs to be addressed is *Aspergillus* resistance. The previously mentioned anti-fungal agents are not active against all *Aspergillus* spp. [13]. Some species may display intrinsic resistance to azoles and polyenes [13,110], while others have acquired resistance to azoles [13,111]. For instance, *Aspergillus calidoustus* and *Aspergillus terreus* exhibit intrinsic resistance to azoles and AMB, respectively [13,110], whereas *Aspergillus fumigatus* may develop acquired resistance to azoles [13,112]. However, anti-fungal susceptibility testing is not recommended for the routine management of the initial infection [2]. More specifically, in patients naïve to azoles or in regions with no documented resistance, anti-fungal susceptibility testing should not be performed [13]. However, such testing is indicated in cases that do not respond to initial treatment, or if there is clinical suspicion of azole resistance [13]. In patients with infections due to *Aspergillus fumigatus* with documented azole-resistance, a group of experts recommended a switch to LAMB or a combination of voriconazole with an echinocandin [112,113]. In cases of environmental resistance to azoles, the latter regimen switch is recommended only if the respective rates are >10% [13]. Azole resistance of *Aspergillus fumigatus* has also been reported in pediatric patients and should be taken into consideration in cases that are unresponsive to azole treatment [114].

#### 3.3.6. Adjunctive Measures

Apart from anti-fungal drugs, treatment for proven/probable IA also includes several adjunctive measures. Colony-stimulating factors (CSFs) may be administered either as prophylaxis, to reduce the duration of granulocytopenia, or as therapy for patients with IA and neutropenia [2]. Granulocyte transfusions may be an option in cases of severe and prolonged neutropenia [8]. An updated Cochrane review, which evaluates the efficacy and safety of this method in the setting of prophylaxis, concluded that the risk of fungemia decreased (low-grade evidence) due to the transfusions, but data was inadequate to support any differences in infection-associated mortality or adverse events [115]. Recombinant interferon gamma (IFN-γ) may be used in cases of severe or refractory IA [2]. Lastly, adoptive transfer of pathogen-specific T lymphocytes, which have derived from donors, is under study for use in IA [85,116,117].

Surgical treatment is reserved for cases of accessible localized lesions [2]. Indications include sinus aspergillosis, localized cutaneous aspergillosis, CNS aspergillosis, pulmonary disease that is localized or adjacent to great vessels/pericardium, or has invaded the pleural space and chest wall, or has caused uncontrolled bleeding [2]. Surgery is contraindicated for unstable patients, or for patients with disseminated disease [6]. In each case, decisions should be individualized [2].

#### 3.3.7. Management of Selected Localized Infections

The following part of this review will be devoted to the management of IPA and CNS aspergillosis in pediatric patients with leukemia and HSCT. These clinical manifestations represent the most common site of infection [6] and one of the most serious complications [118], respectively.

Invasive pulmonary aspergillosis, when suspected, should prompt early initiation of treatment, due to the fact that this practice restricts the development of the disease and due to the unreliability of diagnostic testing [2,89,119]. When the diagnosis is established, the patient should be further evaluated for other foci of IA, such as the CNS [13]. Optimal duration of treatment has not been identified, but a minimum of six to 12 weeks should be applied [2]. Lastly, surgical intervention is reserved for cases with lesions adjacent to great vessels, or vital organs, lesions associated with unmanageable hemoptysis, or lesion causing bone erosions [2].

Aspergillosis of the CNS is most frequently associated with hematologic cancer since the underlying condition in patients >1 year of age [120]. Infection of the CNS has been observed in 6% of children with leukemia with IA [8,121]. The advent of AMB lipid formulations and anti-mold azoles and the progress achieved in early diagnosis have reduced mortality rates from 82.8% to 39.5%, before and after 1990, respectively [121]. Treatment principles of CNS aspergillosis are early diagnosis, initiation of appropriate anti-mold drugs, evaluation of the indications for surgery and decreasing immunosuppression [2,122]. The cornerstone of pharmacotherapy in children has been AMB, with the deoxycholate form having been tolerated in ages <3 months [15,123] and the lipid formulations of AMB having been reserved for older children [15]. Currently, voriconazole is the established first-line treatment of CNS aspergillosis [5,8,81,83,121,124] and the next choice is LAMB at high doses (≥5mg/kg/24 h) [5,125]. TDM for voriconazole is necessary and younger children may require higher doses to reach therapeutic levels [121,126]. However, there is limited evidence regarding the levels of voriconazole in the CSF [121]. Data is also scarce about the use of adjunctive immunotherapy (CSFs, cytokines) in pediatric patients with CNS aspergillosis [120]. Surgical intervention may be indicated in patients with localized lesions [120]. Lastly, the use of corticosteroids and the intrathecal administration of anti-fungals are not recommended [2,127].

## 4. Conclusions

It has become evident that IA is a major issue in immuno-compromised pediatric patients, especially in those with leukemia and in HSCT recipients. Management of this infection consists of two main components, which includes prevention and treatment. The role of primary anti-fungal prophylaxis is highlighted particularly due to the insufficiency of diagnostic tests. Several agents have been evaluated in this setting including triazoles, polyenes, and echinocandins. Empiric and pre-emptive treatments are two approaches that can be initiated before establishing a definitive diagnosis. The mainstay of targeted treatment is voriconazole for children older than two years of age and LAMB in the younger age group. Further research is required in the field of pediatric IA management in order to reach the evidence quantity and quality of the respective field in adults.

## Figures and Tables

**Table 1:** Differences between pediatric and adult Invasive Aspergillosis.

Field of Difference
A) Comorbidities - Biology - Management - Prognosis
B) High-risk populations
C) Epidemiology
D) Diagnostic techniques - Performance - Utility
E) Anti-fungal drugs - Pharmacology - Dosing scheme
F) Phase 3 clinical trials

**Table 2:** Risk factors for Invasive Aspergillosis in pediatric patients.

Leukemia Patients	HSCT Recipients
Severe and persistent neutropenia	Severe and persistent neutropenia
Corticosteroids in high-doses	Corticosteroids in high-doses
Mucosal damage	Mucosal damage
Increasing age	Increasing age
AML	Allogeneic transplant
ALL: relapse	GVHD
ALL: de novo	HLA discordance
ALL: high-risk	CMV coinfection
Refractoriness of acute leukemia	Respiratory virus coinfection
	Colonization by *Aspergillus* spp.
	T-cell depletion
	CD 34 selection
Ward-associated factors (local epidemiology, environmental conditions, contamination of hospital water supply systems, construction works)	Ward-associated factors (local epidemiology, environmental conditions, contamination of hospital water supply systems, construction works)

AML, acute myelogenous leukemia. ALL, acute lymphoblastic leukemia. HSCT, hematopoietic stem cell transplantation. GVHD, graft-versus-host disease. HLA, human leukocyte antigen. CMV, cytomegalovirus. References are provided in the text.

**Table 3:** Primary prophylaxis against Invasive Aspergillosis.

Drug	Route	Dosage	Indications for Use (Recommendation Ranking)	Refs
**AMB formulations**				
LAMB	IV	1 mg/kg/48 h, or 2.5 mg/kg two times per week	High-risk acute leukemia patients (B-II) Allogeneic HSCT recipients (C-III) GVHD (no grading)	[8]
LAMB	Aerosolized	Not established	High-risk acute leukemia patients (no grading) Allogeneic HSCT recipients (no grading)	[8]
**Azoles**				
ITC	PO	2.5 mg/kg/24 h	High-risk acute leukemia patients (B-I) Allogeneic HSCT recipients (B-I) GVHD (C-II)	[8]
VRC	PO IV	9 mg/kg/12 h (ages 2-<12, or 12–14 weighing <50 kg) 200 mg/12 h (ages ≥15 years, or 12–14 weighing ≥50 kg) 9 mg/kg/12 h the first day, followed by 8 mg/kg/12 h on next days (ages 2-<12, or 12–14 weighing <50 kg) 6 mg/kg/12 h the first day, followed by 4 mg/kg/12 h on next days (ages ≥15 years, or 12–14 weighing ≥50 kg)	High-risk acute leukemia patients (no grading) Allogeneic HSCT recipients (B-I) GVHD (B-I)	[8]
PSC	PO	200 mg/8 h (oral susp.) 300 mg/24 h (tabl.)	High-risk acute leukemia patients (B-I) Allogeneic HSCT recipients (no grading) GVHD (B-I)	[8]
**Echinocandins**				
MFG	IV	1 mg/kg/24 h (max 50 mg if weight ≥50 kg) 2 mg/kg/24 h	High-risk acute leukemia patients (no grading) Allogeneic HSCT recipients (C-I) GVHD (no grading) Allogeneic HSCT recipients	[8,61]

AMB, amphotericin B. LAMB, liposomal amphotericin B. IV, intravenous. HSCT, hematopoietic stem cell transplantation. GVHD, graft-versus-host disease. ITC, itraconazole. PO, per os. VRC, voriconazole. PSC, posaconazole. susp., suspension. tabl., tablets. MFG, micafungin.

**Table 4:** Anti-fungal drugs for proven/probable Invasive Aspergillosis.

**Drug**	**Route**	**Dosage**	**Indications for Use (Recommendation Ranking)**	**Refs**
**AMB formulations**				
LAMB	IV	3 mg/kg/24 h ≥5 mg/kg/24 h	First-line treatment (B-I) (especially for ages <2 years) Second-line treatment (B-I) (for cases with VRC intolerance, or for settings with azole resistance) Second-line treatment for CNS Aspergillosis	[1,8,13] [5,81]
ABLC	IV	5 mg/kg/24 h	First-line treatment (B-II) Second-line treatment (B-II)	[8]
**Azoles**				
ITC	PO	2.5 mg/kg/12 h (for ages ≥2 years)	Second-line treatment (no grading) (not approved for ages <18 years)	[8]
VRC	PO IV	9 mg/kg/12 h (ages 2-<12, or 12–14 weighing <50 kg) 200 mg/12 h (ages ≥15 years, or 12–14 weighing ≥50 kg) 9 mg/kg/12 h the first day, followed by 8 mg/kg/12 h on next days (ages 2-<12, or 12–14 weighing <50 kg) 6 mg/kg/12 h the first day, followed by 4 mg/kg/12 h on next days (ages ≥15 years, or 12–14 weighing ≥50 kg)	First-line treatment (A-I) (not approved for ages <2 years) Second-line treatment (A-I)	[8]
PSC	PO	800 mg/24 h divided in 2–4 doses (oral susp.)	Second-line treatment (no grading) (not approved for ages <18 years by the EU, approved by the FDA for ages ≥13 years)	[2,8]
ISA	IV PO	Not established	Evaluation of PK for ages 1–18 years (ClinicalTrials.gov NCT03241550) Evaluation of PK for ages 6–18 years (ClinicalTrials.gov NCT03241550)	[50]
**Echinocandins**				
CAS	IV	70 mg/m^2^ loading dose the first day, followed by 50 mg/m^2^/24 h the next days (maximum 70 mg)	Second-line treatment (A-II)	[8]
MFG	IV	2–4 mg/kg/24 h (100–200 mg/24 h if patient’s weight ≥50 kg)	Second-line treatment (no grading) (non-approved indication by the EU, approved by the FDA for ages ≥4 months)	[1,2,8]
AFG	IV	3 mg/kg loading dose, followed by 1.5 mg/kg/24 h	Not approved by the FDA for ages <18 years	[2,50,82]

AMB, amphotericin B. LAMB, liposomal amphotericin B. IV, intravenous. VRC, voriconazole. CNS, central nervous system. ABLC, amphotericin B lipid complex. ITC, itraconazole. PO, per os. PSC, posaconazole. susp., suspension. EU, European Union. FDA, Food and Drug Administration. ISA, isavuconazole. PK, pharmacokinetics. CAS, caspofungin. MFG, micafungin. AFG, anidulafungin.
